# CAPN1 is a novel binding partner and regulator of the tumor suppressor NF1 in melanoma

**DOI:** 10.18632/oncotarget.25805

**Published:** 2018-07-27

**Authors:** Michal Alon, Rand Arafeh, Joo Sang Lee, Sanna Madan, Shelly Kalaora, Adi Nagler, Tereza Abgarian, Polina Greenberg, Eytan Ruppin, Yardena Samuels

**Affiliations:** ^1^ Molecular Cell Biology Department, Weizmann Institute of Science, Rehovot, Israel; ^2^ Center for Bioinformatics and Computational Biology, The University of Maryland, College Park, Maryland, USA; ^3^ Cancer Data Science Lab, National Cancer Institute, National Institute of Health, Bethesda, Maryland, USA

**Keywords:** melanoma, NF1, CAPN1, degradation, combination therapy

## Abstract

Neurofibromin 1 (NF1), a tumor suppressor that negatively regulates RAS through its GTPase activity, is highly mutated in various types of sporadic human cancers, including melanoma. However, the binding partners of NF1 and the pathways in which it is involved in melanoma have not been characterized in an in depth manner. Utilizing a mass spectrometry analysis of NF1 binding partners, we revealed Calpain1 (CAPN1), a calcium-dependent neutral cysteine protease, as a novel NF1 binding partner that regulates NF1 degradation in melanoma cells. ShRNA-mediated knockdown of CAPN1 or treatment with a CAPN1 inhibitor stabilizes NF1 protein levels, downregulates AKT signaling and melanoma cell growth. Combination treatment of Calpain inhibitor I with MEKi Trametinib in different melanoma cells is more effective in reducing melanoma cell growth compared to treatment with Trametinib alone, suggesting that this combination may have a therapeutic potential in melanoma. This novel mechanism for regulating NF1 in melanoma provides a molecular basis for targeting CAPN1 in order to stabilize NF1 levels and, in doing so, suppressing Ras activation; this mechanism can be exploited therapeutically in melanoma and other cancers.

## INTRODUCTION

Melanoma is the deadliest form of human skin cancer for which the incidence rate continues to increase [1]. In recent years, the genetic landscape of melanoma has been extensively studied [2–4], which has enabled the development of highly effective targeted therapies [5–7]. However, not all patient tumors are amenable to these treatments, with those tumors that are not eradicated by the treatment have been found to rapidly develop resistance [8–12]. A better understanding of these pathways in which tumor drivers function is essential. Unraveling the mechanisms by which cancer cells become resistant to drugs and developing new agents to target the relevant pathways represent the next logical steps in personalized cancer treatment. One approach to tackle these challenges in melanoma is to characterize the drivers’ protein interactions by unbiasedly investigating driver protein's binding partners. Importantly, these proteomic investigations might provide novel molecules that can serve as biomarkers for cancer diagnosis, prognosis or therapy [13, 14].

On the basis of exome and genome sequencing studies, cutaneous melanoma is divided into four different molecular subgroups: The first group includes *BRAF* (most often *BRAF^V600E^*) mutant melanomas, the second group are *NRAS^Q61L/R^* mutant melanomas, the third group are *NF1* mutant melanomas, and the fourth group are triple wild-type melanomas [2]. *NF1,* which is mutated in 14% of melanoma patients, is a tumor suppressor gene that encodes a RAS GTPase activating protein (RAS GAP), which negatively regulates RAS by catalyzing the hydrolysis of RAS-GTP to RAS-GDP [15]. Germline mutations in *NF1* drive neurofibromatosis type I, a familial cancer syndrome affecting one in 3,500 individuals worldwide. Neurofibromatosis patients suffer from benign neurofibromatosis, malignant sarcomas, gliomas, pheochromocytomas, gastrointestinal stromal tumors and myeloid leukemia [16, 17]. Further, the *NF1* gene is frequently mutated in various types of sporadic human cancers, including glioblastoma [18], neuroblastoma [19], acute myeloid leukemia [20], as well as lung [21], ovarian [22] and breast tumors [23], thus highlighting a broader role for *NF1* in human cancer.

Neurofibromin1 is best acknowledged as a RasGAP [24, 25]. However, this function is enabled only by a small part (~13%) of this large protein (2800 amino acids) [24]. The function and the structure of most of the NF1 domains is not fully characterized. Furthermore, although it has been reported that NF1 is regulated by the ubiquitin-proteasome system in response to a variety of growth factors through the activation of protein kinase C [26–28], the molecular mechanisms underlying NF1 regulation are still not entirely understood. In addition to its RasGAP domain, NF1 contains multiple other domains, including a cysteine-serine rich domain (CSRD), tubulin binding domain (TBD), SEC14 domain, pleckstrin homology (PH) domain, carboxy-terminal domain (CTD) and syndecan-binding domain (SBD). Several of the proteins shown to associate with NF1 are involved in cellular processes such as intracellular trafficking (Tubulin, APP, LRPPRC, Kinesin 1), neural differentiation (VCP, DPYSL2), membrane localization (Syndecan, Caveolin 1, SPRED1), actin cytoskeleton remodeling (LIMK2), ubiquitylation (Cullin 3, SCF, FAF2), cell adhesion (FAK) and cell signaling (DDAH1, 14-3-3). Some proteins such as Kinesin 1, Cullin 3, Caveolin 1 and SPRED1 were shown to interact with the NF1 domain, but their binding site is as of yet unknown [17, 29]. Identification of these interacting proteins was based on binding to a specific domain of NF1 rather than to the full protein and also by using a variety of biochemical approaches, including the yeast two hybrid system, rather than physiological systems. In addition, the biological significance of these interactions is still not fully understood and these NF1 binding partners were not demonstrated in melanoma.

In this study, we applied a mass-spectrometry approach to identify novel NF1 binding partners in different melanoma cell lines. We identified Calpain1 (CAPN1), a calcium-dependent neutral cysteine protease as a novel NF1 binding partner. Calpains are part of a regulatory proteolytic system responsible for the degradation of membrane and cytoskeletal proteins, kinases, phosphatases and transcription factors [30]. The two isoforms of the ubiquitous calpain, μ-calpain (CAPN1) and m-calpain (CAPN2), differ mainly in the calcium concentration needed for their activation (μM and mM, respectively). CAPN1 and CAPN2 are heterodimers of a large catalytic (80 kDa) subunit (encoded by *CAPN1* and *CAPN2*, respectively) and a regulatory subunit (28 kDa), which is common to both isoforms and is encoded by *CAPNS1* [31]. In this study, we show that CAPN1 regulates NF1 protein expression levels and as a consequence regulates RAS activity. Thus, in addition to the identification of a new RAS regulatory factor, our findings also reveal a novel strategy for suppressing RAS activation, which may have a therapeutic potential.

## RESULTS

### NF1 and CAPN1 are novel binding partners

To further characterize NF1 functional interactions, we conducted a mass spectrometry-based screen. Mass spectrometry was performed on endogenous NF1 co-immunoprecipitates that were generated from two melanoma cell lines: A375, a commercial melanoma cell line, and 74T, a cell line derived from a melanoma patient. Both cell lines are *NF1* wild-type, with A375 also containing a *BRAF^V600E^* mutation and 74T, an *NRAS^Q61K^* mutation, which allowed us to screen for NF1 interactions in the two major mutational backgrounds of melanoma [2].

This approach led to the identification of 167 and 139 NF1 unique peptides in the A375 and 74T cell lines, respectively. The analysis revealed A375 to carry eight unique peptides of SPRED2, a known NF1 interacting protein that belongs to the SPRED family, which is responsible for the translocation of NF1 to the plasma membrane [32]. Importantly, we also detected CAPN1 in the NF1 immunoprecipitates of both cell lines. We further identified 11 and 7 unique peptides belonging to the CAPN1 small subunit and 32 and 16 belonging to its catalytic subunit in A375 and 74T, respectively. The mass spectrometry analysis also revealed peptides belonging to Calpastatin, the endogenous inhibitor of CAPN1 [30] (43 and 21 unique peptides in A375 and 74T, respectively). This identification strengthens our finding that CAPN1 is indeed a new NF1 interacting protein. The full list of peptides identified in the mass spectrometry analysis is described in Supplementary Table 1.

**Figure 1:**
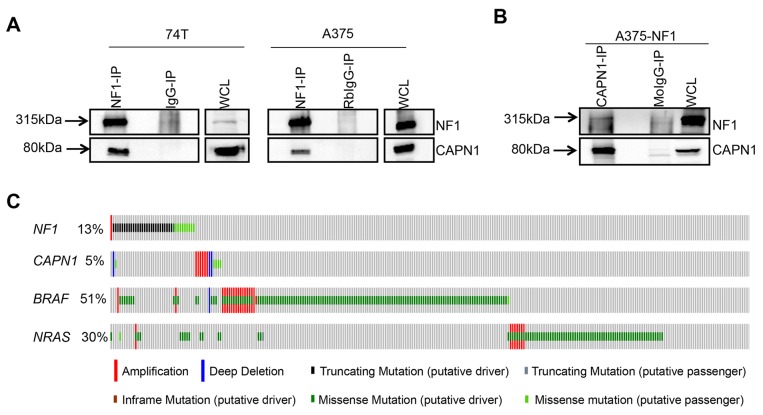
CAPN1 is a novel binding partner of NF1 **(A)** Immunoblots of melanoma cells immunoprecipitated with anti-NF1. Immunoprecipitates were analyzed in parallel by anti-NF1 and anti-CAPN1 immunoblotting. **(B)** Immunoblots of melanoma cells immunoprecipitated with anti-CAPN1. Immunoprecipitates were analyzed in parallel by anti-NF1 and anti-CAPN1 immunoblotting. WCL – whole cells lysate, RbIgG-IP and MoIgG- Rabbit and mouse IgG isotype control, respectively. **(C)** Distribution of alterations in *NF1*, *CAPN1*, *BRAF* and *NRAS* in TCGA melanoma (N=287).

To validate the interaction between NF1 and CAPN1, lysates from A375 and 74T were immunoprecipitated using an anti-NF1 antibody, followed by SDS-PAGE and immunoblotting with anti-CAPN1. We found that endogenous NF1 co-immunoprecipitates with CAPN1 in both cell lines (Figure 1A). When CAPN1 was immunoprecipitated from A375 cells that overexpress NF1, NF1 was also detected (Figure 1B). These results confirm that NF1 and CAPN1 are indeed binding partners.

### Mutual exclusivity analysis of *NF1* mutant melanomas and *CAPN1*

In order to further evaluate whether *NF1* and *CAPN1* interact genetically, we relied on previous studies that had found mutually exclusive mutated genes to be members of the same functional pathway. Specifically, if two genes belong to the same cancer-driving pathway, then a mutation in just one of them may suffice to dysregulate the pathway [33, 34]. Thus, systematic mutual exclusivity can potentially provide important information about unknown functional interactions and alternative cancer progression pathways. We analyzed The Cancer Genome Atlas (TCGA) database to look for mutually exclusive patterns between *NF1* nonsense mutations and *CAPN1*. As can be seen in Figure 1C, the *CAPN1* (11q13.1) locus was amplified in 5% of human melanoma cases, and was consistently retained in a mutually exclusive fashion in *NF1*-deficient melanomas (*P* = 0.024, the FDR corrected *P*-value was calculated using WEXT) [35], further suggesting that they are found in the same functional pathway.

### CAPN1 is involved in NF1 proteolysis

**Figure 2:**
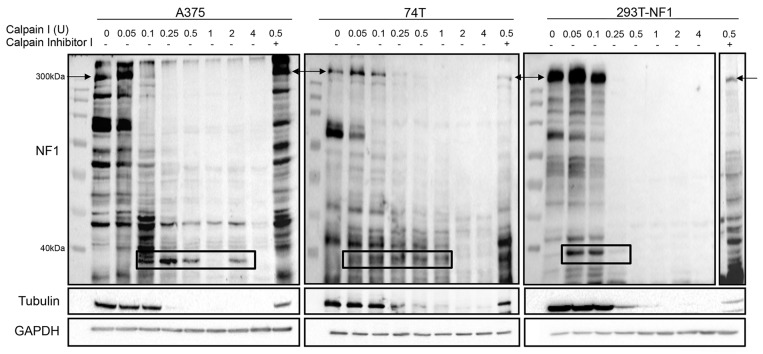
CAPN1 is responsible for NF1 degradation *in vitro* Cell lysates of A375, 74T and 293T stably over expressing NF1 (293T-NF1) were incubated with the indicated units of purified CAPN1 with 10 mM CaCl_2_ for 1 hour, resolved by SDS-PAGE and blotted with anti-NF1 (polyclonal antibody) and anti-GAPDH. Anti-Tubulin served as a positive control for the assay. Molecular weights are given in kilodaltons on the left. The arrows indicate the detection of full length NF1 and the black boxes indicate an approximately 40 kDa proteolytic fragment of NF1. Addition of 45 μM of Calpain inhibitor I blocked this degradation (last lane on each blot).

CAPN1 activation was previously shown to promote the degradation of tumor suppressor gene products, such as p53 and NF2 [36, 37]. As *NF1* is a tumor suppressor gene, we were interested in investigating whether CAPN1 could degrade NF1, which would reveal a new mode of regulation of NF1 in melanoma, in addition to the degradation of NF1 by the ubiquitin-proteasome pathway [26]. Therefore, we treated A375, 74T, and 293T cells stably overexpressing NF1 (293T-NF1) with increasing concentrations of purified CAPN1, and then assessed NF1 protein levels (Figure 2). NF1 degradation levels rose in a dose-dependent manner. The cleavage of NF1 was followed by the appearance of an approximately ~40 kDa proteolytic fragment that was detected mostly in the incubations with the lower concentrations of CAPN1 (0.05 and 0.1 U in 74T and 293T-NF1 and 0.1, 0.25, 0.5 and 2 U in A375). However, this proteolytic product was not detected with higher concentrations of CAPN1 (1- 4 U range, except for the case of 2 U in A375), probably due to full proteolysis by CAPN1 in these concentrations. In addition, NF1 degradation was halted when the cell lysates were treated with Calpain inhibitor I (ALLN), a synthetic tripeptide aldehyde that acts as a potent inhibitor of CAPN1. Tubulin, which is a known CAPN1 substrate [38], served as a positive control and was also found to be degraded in a dose-dependent manner (Figure 2). These findings establish NF1 proteolysis by CAPN1 *in vitro*.

To identify the CAPN1 proteolytic sites in NF1, we purified NF1 from 293T-NF1 cells by Flag immunoprecipitation and elution; the purified NF1 protein was then subjected to CAPN1 degradation and the degradation product was identified by mass spectrometry analysis. We identified 15 NF1 peptides in this analysis, mapped mainly to the last 320 amino acids of the C-terminus (~35.2 kDa) of the NF1 protein (Supplementary Figure 1). We used the novel GPS-CCD (Calpain Cleavage Detector) software package to predict Calpain cleavage sites http://journals.plos.org/plosone/article?id=10.1371/journal.pone.0019001) and found a predicted cleavage site, located at position 2,512 (YLAATYPTVG|QTSP, score -0.823) in the NF1 protein (Supplementary Figure 2). This predicted location is in line with the peptide coverage map that we obtained via the mass spectrometry analysis, i.e., the first peptide detected at the C-terminus starts at 2521 in the NF1 protein. In order to further confirm our findings, we used different NF1 antibodies recognizing distinct epitopes in the NF1 protein (Supplementary Figure 2). We were able to detect the ~40 kDa product only when we blotted with antibodies recognizing the C-terminus of the NF1 protein. Thus, this confirms that the NF1 degradation product is indeed the ~35.2 kDa fragment found in the C-terminus part of the full length NF1 protein, also identified by mass spectrometry.

### CAPN1 inhibition stabilizes NF1 levels and suppresses RAS and AKT signaling

Since NF1 negatively regulates RAS-GTP (i.e., the active RAS) [39], and since we showed that CAPN1 is involved in the degradation of NF1, we next sought to determine the effect of CAPN1 inhibition on the levels of NF1 and on its downstream pathways, i.e., RAS, MAPK and PI3K.

**Figure 3:**
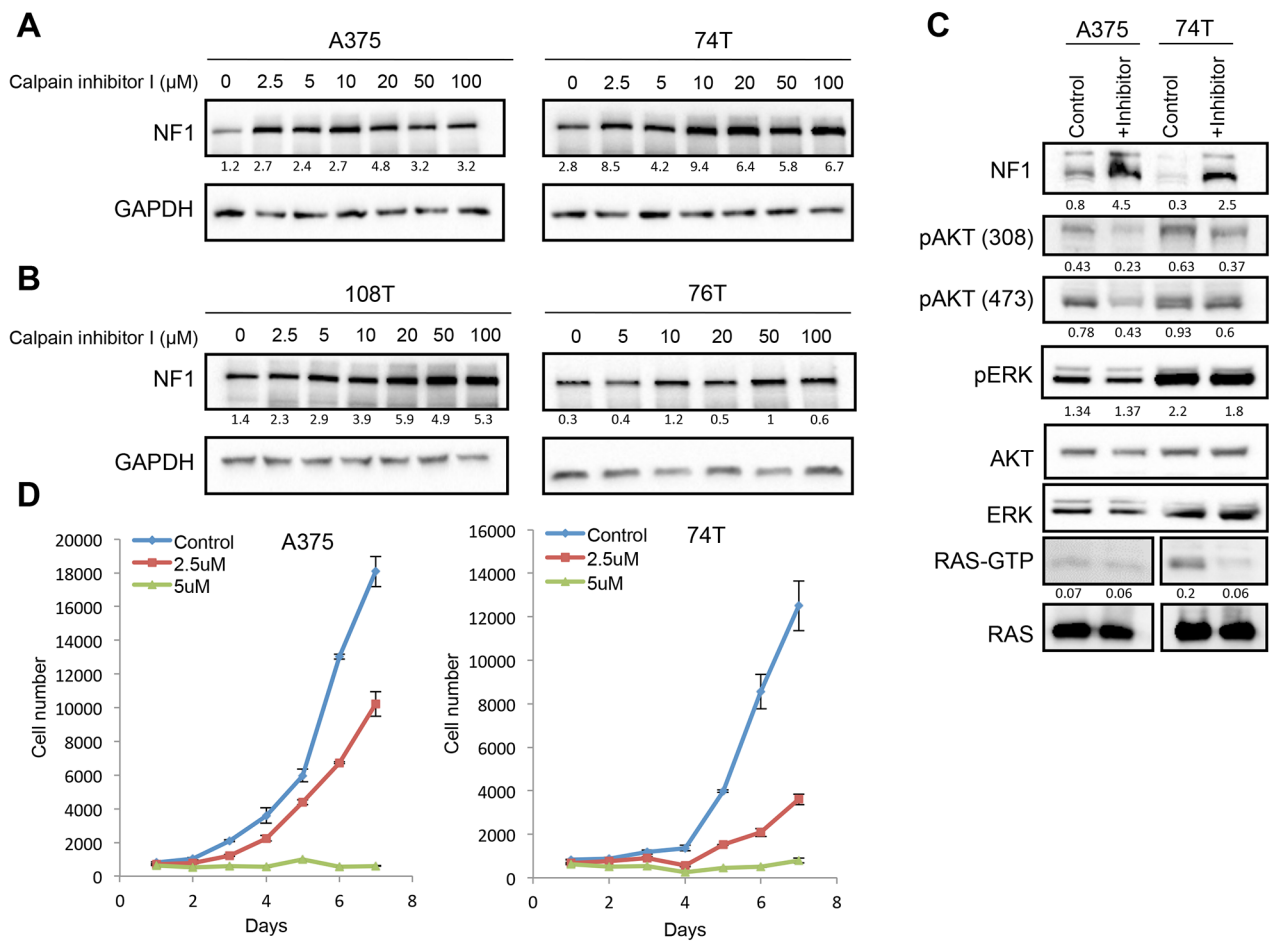
CAPN1 inhibition stabilizes NF1 levels, affecting RAS signaling and cell proliferation **(A)** *NF1* wild type cells (A375, 74T) were treated with increasing concentrations of Calpain inhibitor I (μM) for 6 hours or with DMSO as control, and NF1 levels were tested by immunoblot. **(B)** *NF1* mutant cells (108T, 76T) were treated with increasing concentrations of Calpain inhibitor I (μM) for 6 hours or with DMSO as control, and NF1 levels were tested by immunoblot. Ratios of NF1 levels to GAPDH were generated using Image lab (BioRad) and Microsoft Excel analysis. **(C)** 74T and A375 cells were treated with 3 μM or 5 μM of Calpain inhibitor I for 16 hours, respectively. Cell lysates were analyzed by western blot with the indicated antibodies. RAS-GTP levels were assessed by RAS pulldown assay after treatment with 50 μM of Calpain inhibitor I, respectively for 6 hours. **(D)** A375 and 74T cells were seeded in the 96 well plates with 10% FBS and cells were treatedwith 2.5 or 5 μM of Calpain inhibitor I, respectively. DMSO was used as a control for this experiment. The average cell number was measured by assessing DNA content using SYBR green I in two independent experiments with six replicates each. Error bars, s.e.m.

To determine whether CAPN1 directly affects NF1 stability, we treated A375 or 74T melanoma cells with increasing concentrations of Calpain inhibitor I. A significant increase in NF1 levels was observed following the inhibitory treatment, suggesting that CAPN1 is indeed involved in NF1 stability (Figure 3A). Importantly, NF1 stabilization was also observed in two *NF1* mutant melanoma cells that harbor missense mutations in one of the *NF1* alleles: 108T (p.H1721Q) and 76T (p.P1667S) (Figure 3B). These results indicate that CAPN1 inhibition can stabilize NF1 levels both in wild-type and *NF1* mutant melanoma cells. CAPN1 inhibition did not alter NF1 mRNA levels in A375 and 74T cells (Supplementary Figure 3), suggesting that CAPN1 regulates protein stability rather than the transcription of *NF1*.

As NF1 is a RasGAP, known to inhibit Ras GTPase activity, which activates ERK and AKT pathways and regulates many cellular functions such as cell proliferation, differentiation, apoptosis, and senescence, we next addressed the biological consequences of NF1 stabilization on ERK and AKT pathways. Treatment of A375 and 74T cells with Calpain inhibitor I suppressed AKT signaling by inhibiting AKT-308 and 473 phosphorylation in both cell lines, while ERK signaling was not affected. Inhibition of CAPN1 had a similar suppressive effect on Ras-GTP levels in the 74T cell line. A RAS activation assay showed a 3.6-fold decrease in RAS-GTP levels in 74T cells, but there was no significant change in RAS-GTP levels in A375, probably due to high BRAF^V600E^ activity in these cells, which suppresses RAS (Figure 3C). This is consistent with previous studies showing that above a certain threshold of active protein, RAS brings about maximal MAPK pathway activation [40, 41].

Previous studies reported that inhibition of the RAS and AKT pathways reduces cell proliferation [42, 43]. As NF1 stabilization by CAPN1 inhibition downregulates the AKT pathway, we investigated whether NF1 stabilization also affects cell proliferation. As shown in Figure 3D, treatment of A375 or 74T cells with Calpain inhibitor I significantly reduced their proliferation rate. Thus, CAPN1 inhibition stabilizes NF1 levels, leading to suppression of RAS/AKT signaling and reduction of cell growth.

In order to test the specificity of the CAPN1 inhibition effect in stabilizing NF1 protein levels, decreasing RAS/AKT signaling pathways and reducing cell proliferation in *NF1*-wild type melanoma cells, we tested the effect of CAPN1 inhibition on an *NF1-*null melanoma cell line, CO84, which was derived from a melanoma patient, that harbored the p.R2517^*^ *NF1* truncation mutation. NF1 protein was not detected in CO84 cells. Most importantly, no stabilization in NF1 protein levels occurred after treatment with Calpain inhibitor I (Supplementary Figure 4A). Additionally, there was no change in ERK and AKT signaling (Supplementary Figure 4B).

In addition, we tested the effect of CAPN1 inhibition in a cell line where we knocked down NF1 levels. We used two short hairpin RNA (shRNA) to stably knockdown NF1 in A375 cell line. Immunoblotting confirmed the specific targeting of NF1 by the different shRNAs (Supplementary Figure 4C). When NF1 knockdown cells were treated with Calpain inhibitor I, NF1 was not stabilized and no change was observed in ERK and AKT signaling (Supplementary Figure 4D), therefore demonstrating the direct specificity of Calpain inhibition on the stabilization of NF1 levels and its indirect effects on RAS/AKT pathways.

### Loss of CAPN1 stabilizes NF1 levels and suppresses RAS/AKT signaling

**Figure 4:**
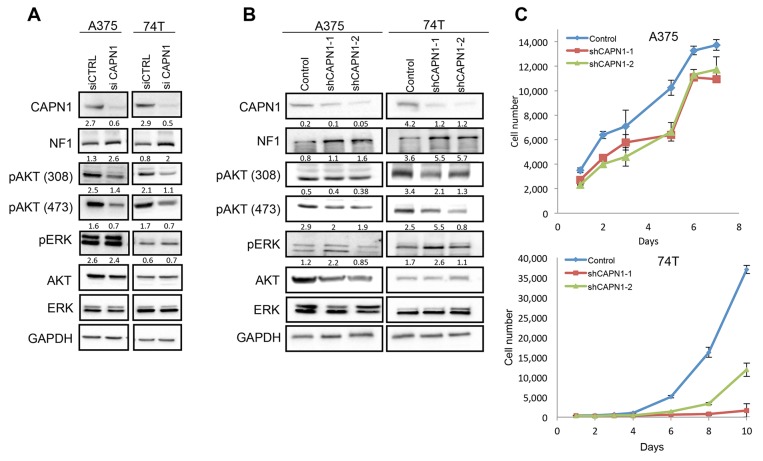
Suppression of CAPN1 by shRNA stabilizes NF1 levels and affects Ras signaling and cell proliferation **(A)** Immunoblot of lysates generated from siRNA mediated *CAPN1* knockdown and control tested by transient transfection of 100 nM for 72 hours. **(B)** Immunoblots of lysates generated from two shRNA mediated *CAPN1* knockdown (shCAPN1-1 and shCAPN1-2) compared to the control vector. Quantification values are given under the blots generated by Image lab (BioRad) and Microsoft Excel analysis. **(C)** 74T and A375 cells stably expressing shRNA against *CAPN1* (shCAPN1-1 or shCAPN1-2) were grown in 96 well plates with 10% or 2.5% FBS, respectively. The average cell number was measured by assessing DNA content using SYBR green I in two independent experiments with six replicates each. Error bars, s.e.m.

To assess whether the loss of CAPN1 in melanoma cells could also stabilize NF1 levels and reduce melanoma cell proliferation, we used small interfering RNA (siRNA) to deplete CAPN1 in both A375 and 74T melanoma cells. We confirmed the specific targeting of CAPN1 by siRNAs in both cell lines by immunoblotting. We found that CAPN1 knockdown stabilized NF1 protein levels and suppressed AKT signaling, by downregulating phosphorylation of AKT-308 and AKT-473 in A375 and 74T cells, whereas the levels of ERK activation remained unchanged (Figure 4A). Next, we tested the effect of CAPN1 knockdown on melanoma cell proliferation. For this, we used two short hairpin RNA (shRNA) to stably knockdown the CAPN1 protein in the A375 and 74T cell lines. Immunoblotting confirmed the specific targeting of *CAPN1* by shRNAs, NF1 stabilization, as well as a reduction in pAKT-308 and pAKT-473 in both melanoma cell lines, while ERK activation remained unchanged (Figure 4B). *CAPN1*-specific shRNA substantially reduced the growth of 74T cells compared to control cells. In A375 cells, the reduction in growth rate was milder, probably due to the presence of the *BRAF^V600E^* mutation [44] (Figure 4C). NF1 stabilization, suppression in AKT activation and reduction in cell proliferation are consistent with the effects obtained after CAPN1 inhibition (Figure 3). Taken together, these data show that CAPN1-mediated NF1 degradation plays an essential role in regulating RAS and AKT pathway and that CAPN1 suppression inhibits this pathway.

**Figure 5:**
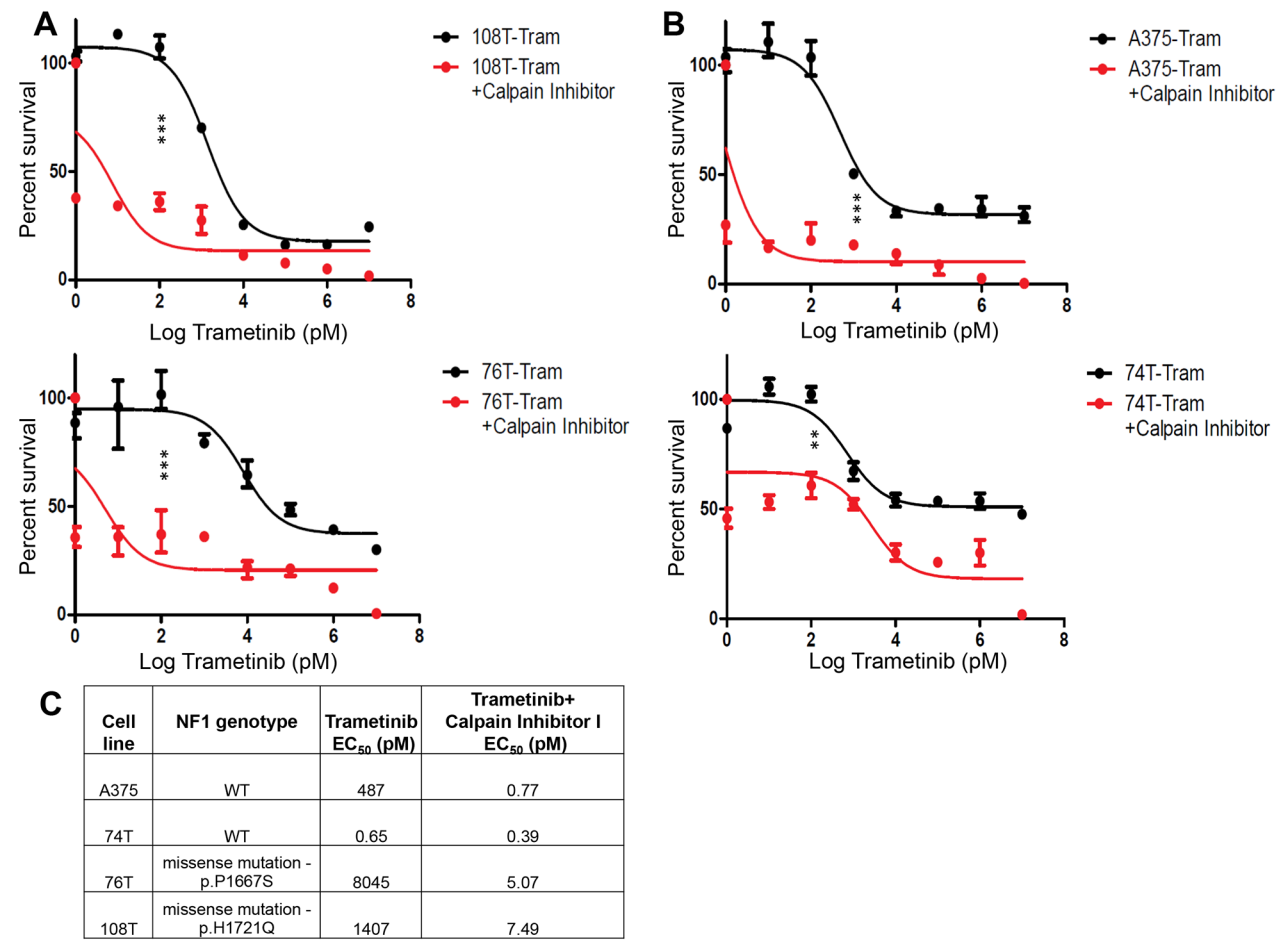
*NF1* mutant and wild-type cell lines show enhanced sensitivity to combined CAPN1 and MEKi inhibition **(A)** Representative dose response curves were generated using *NF1* mutant cell lines (108T and 76T) treated with constant concentration of Calpain inhibitor I (6 μM) and increasing concentrations of Trametinib (1 pM - 10 μM) for 72 hours before assessing viability by Cell Titer-Glo Luminescent Cell viability assay (n=3). The relative cell number after cells were treated with Calpain inhibitor I and Trametinib is plotted as percent survival, as compared to Trametinib-treated control, versus log Trametinib concentration in pM. **(B)** Representative dose response curves were generated using *NF1* wild-type cell lines (A375 and 74T) treated with constant concentration of Calpain inhibitor I (6 μM and 4 μM, respectively) and increasing concentrations of Trametinib (1 pM - 10 μM) for 72 hours before assessing viability by Cell Titer-Glo Luminescent Cell viability assay (n=3). The relative cell number after cells were treated with Calpain inhibitor I and Trametinib is plotted as percent survival, as compared to Trametinib-treated control, versus log Trametinib concentration in pM. ^**^P<0.01 and ^***^P<0.0001 Trametinib versus Trametinib and Calpain inhibitor I (Student *t* test). **(C)** EC_50_ values for inhibition of cell growth by 72 hours treatment with Trametinib or with the combination of Trametinib and Calpain inhibitor I.

NF1 loss was previously shown to drive resistance to many different drugs in melanoma [45, 46]. We therefore sought to determine whether NF1 restoration and stabilization by inhibiting CAPN1 could affect sensitivity to pharmacologic MEK inhibition. In particular, we hypothesized that inhibition of MEK and upregulation of NF1 levels, by CAPN1 inhibition, might have a synergistic effect on melanoma cells. Here, combined inhibition of MEK and CAPN1 by simultaneous treatment with Trametinib and Calpain Inhibitor I achieved greater efficacy than Trametinib alone both in wild-type *NF1* melanoma cells (A375 and 74T) and in melanoma cells that harbor one *NF1* mutant allele and another wild-type one (108T and 76T) (Figure 5). Since we showed that CAPN1 inhibition did not stabilize NF1 in the truncated *NF1* mutant cells (CO84) or in *NF1* null cells (A375 with shRNA against NF1) (Supplementary Figure 4A and 4D), we conclude that, in 108T and 76T, where one *NF1* allele is mutated, the stabilization of wild-type NF1 is sufficient to decrease Ras/AKT signaling and cell proliferation. The fact that CAPN1 inhibition has growth inhibitory effects on *NF1* wild-type and as well on *NF1* mutant melanoma cell lines could have a beneficial therapeutic potential.

### Alterations in NF1 and CAPN1 affect the overall survival of melanoma patients

**Figure 6:**
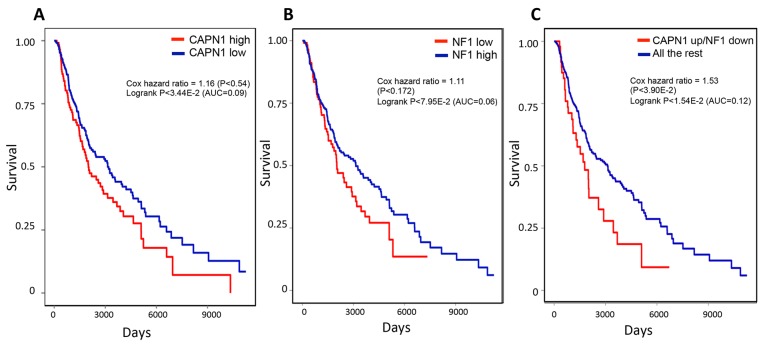
Kaplan-Meier plots of melanoma patients with CAPN1 over expression and NF1 under expression show worse prognosis compared to patients with alterations only in *CAPN1* or in *NF1* **(A)** Survival plot of patients with CAPN1 over expression or missense mutation in TCGA melanoma (N=355). **(B)** Survival plot of patients with NF1 under expression or missense mutation in TCGA melanoma (N=355). **(C)** Survival plot of patients with CAPN1 over expression or missense mutation and NF1 under expression or missense mutation in TCGA melanoma (N=355). A gene in a sample was marked under (over) expressed if its expression level in the sample is below 1/3-quantile (above 2/3-quantile) of its expression level across TCGA melanoma samples. Each association was evaluated using Cox proportional hazard model while controlling for patients’ age, sex, and race, tumors’ genomic instability and tumor stage (see Methods).

Given that Calpain inhibitor I expression leads to increased NF1 stability and RAS inhibition and the strong contribution of the RAS pathway to promoting tumorigenesis [47], we reasoned that NF1 and CAPN1 expression levels may affect melanoma patient survival. We, therefore, analyzed the overall survival of a cohort of 355 TCGA melanoma patients. Patients were grouped according to the presence and absence of missense *NF1* and *CAPN1* mutations, as well as their expression levels, which were calculated based on each sample's expression data and somatic mutations data (see Methods section). We found that neither alteration in *NF1* nor in *CAPN1*, as a single component, had an effect on patient survival (Figure 6A and 6B). However, patients with high CAPN1 and low NF1 expression levels had a worse prognosis (Figure 6C). These conclusions also hold when controlling for potential confounding factors, including age, tumor stage and genomic instability (see Methods section), testifying to the synergistic effect in the tumors, where the enhanced CAPN1-induced degradation of the already downregulated NF1 protein results in aggressive tumor growth and poor survival.

## DISCUSSION

The RAS pathway plays an important role in different complex cellular processes such like proliferation, differentiation, transformation, development and apoptosis [39]. The intensity of the RAS/ERK/AKT signaling directs these cellular processes. Not much is known about the molecular mechanims that regulate the intensity of the RAS/ERK/AKT signaling. Here, we report that CAPN1 regulates the degradation of the tumor suppressor NF1, a negative regulator of Ras.

Although RAS was discovered to be a major oncogene that is mutated in many human tumors more than 30 years ago, finding an inhibitor that is selective for oncogenic RAS remains one of the major challenges in cancer therapy. Despite decades of research on RAS, it is still undruggable [47, 48]. In this study, we have found an indirect way to inhibit RAS activity. CAPN1 suppression potently inhibits RAS/AKT activation and cell proliferation through its effects on NF1 (Model found in Supplementary Figure 5).

Since NF1 is highly mutated in melanoma and various other cancer types [49], it is evident that this tumor suppressor plays an important role in cancer development and progression. Therefore, an in-depth understanding of its mode of regulation could provide significant insights into its function in different cell types and, consequently, better understanding of the consequences of its loss. Here, we reveal CAPN1 to be a novel NF1 regulatory factor capable of degrading NF1. Furthermore, CAPN1 suppression inhibits RAS/AKT activation and impedes cellular proliferation by stabilizing NF1 levels (Supplementary Figure 5). Thus, these findings demonstrate a new regulatory component of the RAS pathway and provide new insights as to how RAS activity can be controlled. Furthermore, CAPN1's ability to regulate NF1 and RAS may be utilized to suppress tumor development. Based on these observations, CAPN1 inhibitors are expected to suppress tumors in which NF1 is destabilized. Over the past three decades at least 60 calpain inhibitors derived from both natural sources and chemical synthesis have been developed and screened in various models. One of the inhibitors, developed by Abbot Pharmaceuticals, is under phase I clinical trial to treat Alzheimer disease (A-705253, ABT-957) and another compound (CAT811) presented promising results in animal models for treatment of cataracts [50, 51].

In melanoma, calpain inhibition limits tumor growth *in vitro* and *in vivo*, but increases dissemination by amplifying cell resistance to apoptosis and accelerating migration process [52]. In addition, inhibition of Calpain by either Calpain inhibitor I (ALLN) or Calpastatin (CS) peptide blocked melanin biosynthesis in mouse B16 melanoma cells, which was correlated with a decrease in the activity of tyrosinase, a key regulatory enzyme in melanogenesis [53].

Aberrant expression of Calpain has been implicated in tumorigenesis. Increased expression of CAPN1 has been observed in schwannomas and meningiomas [37] and heightened expression of CAPN2, in colorectal adenocarcinoms [54]. In addition, Calpains were found to promote oncogenesis by degrading tumor suppressor genes, such as p53 or NF2 [36, 37]. Here, we show that the NF1 tumor suppressor is an additional substrate of CAPN1.

The RAS/MAPK/AKT signaling pathways are a major target for cancer therapy, however the presence of *NF1* mutations, which results in reduced expression of NF1, confers resistance to several therapeutic targets. *NF1* loss has been shown to drive resistance to RAF inhibitor, dasatinib, tamoxifen and retinoic acid in melanoma [25], lung cancer [55], breast cancer [56] and neuroblastoma [19], respectively. We found that when melanoma cells harboring wild-type *NF1* alleles or melanoma cell lines with a heterozygous *NF1* mutation in one allele and the other allele is intact were treated with combination of Calpain inhibitor I and Trametinib, a clear synergistic effect was observed. Inhibition of growth was significantly higher in the combination treatment compared to treatment with Trametinib alone. This combination might therefore have a therapeutic potential not only in melanoma cells harboring wild type *NF1* alleles, but also in melanoma cells harboring only one *NF1* wild type allele.

Given the importance of the RAS/AKT pathways in cancer, our findings should provide the molecular model and the rationale for developing CAPN1 inhibitors that halt NF1 destruction and, therefore, block RAS activation, downregulate AKT signaling, which may be useful as a therapeutic strategy in melanoma, and possibly also in other cancers, where NF1 expression is destabilized. These data also provide exciting new opportunities for combination therapies.

## MATERIALS AND METHODS

### Tumor tissues

All melanoma cell lines were established as described previously [57]. A subset of cell lines used in the study (74T, 76T, and 108T) were derived from a panel of pathology-confirmed metastatic melanoma tumor resections collected from patients enrolled in institutional review board (IRB)-approved clinical trials at the Surgery Branch of the National Cancer Institute. Pathology-confirmed melanoma cell lines were derived from mechanically or enzymatically dispersed tumor cells, which were then cultured in RPMI-1640 supplemented with 10% FBS, L-glutamine, penicillin and streptomycin at 37°C in 5% CO_2_ for 5–15 passages. A375 cells were obtained from the American Type Culture Collection (ATCC).

### Pooled stable expression

To produce lentivirus, the NF1 construct in which NF1 is tagged with Flag-HA at the N-terminus (pHAGE-NF1, kind gift from Karen Cichowski, BWH Brigham Research Institute) was co-transfected into HEK293T cells seeded at 2.5×10^6^ per T75 flask with pVSV-G and pHRCMV-8.2ΔR helper plasmids using Lipofectamine2000 as described by the manufacturer. Virus-containing media was harvested 60 hours after transfection, filtered, aliquoted and stored at -80°C. Lentivirus for NF1 were used to infect the A375 and HEK293T cells as previously described [40]. Stable expression of NF1 was determined by SDS-PAGE analysis followed by immunoblotting with polyclonal anti-NF1 (A300-140A; Bethyl) and anti-Flag (M2) (F7425, Sigma) to show equivalent expression among pools.

### Identification of NF1 interacting proteins

Cells were gently washed in PBS trypsinized and then lysed using lysis buffer (1% NP-40, 50 mM Tris-HCl pH 7.5, 150 mM NaCl, 0.5% deoxycholic acid, 1% complete protease inhibitor (Roche), 1 mM sodium orthovanadate, 1 mM sodium fluoride and 0.1% SDS. Lysates were incubated for 15 min on ice and then centrifuged for 15 min at 13,000 rpm at 4°C. 3 mg of the protein lysates were taken for immunoprecipitation of NF1 or a control reaction with protein A/G beads (Santa Cruz) with 2 μg of NF1 antibody or normal rabbit immunoglobin G (IgG) (Dako), in rotation overnight at 4°C. Immunoprecipitations were washed five times with lysis buffer then resuspended with sample buffer before denaturation and separation by SDS-PAGE on 8% mini gels. The proteins in the gel were visualized with Imperial™ protein stain (Thermo Scientific), then reduced with 3 mM DTT (60°C for 30 min), modified with 10 Mm iodoacetamide in 100 mM ammonium bicarbonate (in the dark, room temperature for 30 min) and digested in 10% acetonitrile and 10 mM ammonium bicabonate with either modified trypsin or chymotrypsin (Promega) at a 1:10 enzyme-to-substrate ratio, overnight at 37°C. Alternatively, proteins mixture in 8 M Urea and 100 mM ammonium bicarbonate were reduced and modified as described and digested in 2 M Urea, 25 mM ammonium bicabonate with modified trypsin or chymotrypsin (Promega) at a 1:50 enzyme-to-substrate ratio. The resulted peptides were desalted using C18 tips (Homemade stage tips) and were subjected to LC-MS-MS analysis. The peptides were resolved by reverse-phase chromatography on 0.075 × 180-mm fused silica capillaries (J&W) packed with Reprosil reversed phase material (DrMaisch GmbH, Germany). The peptides were eluted with linear 30 min gradient of 5% to 35% acetonitrile with 0.1% formic acid in water, 15 min gradient of 35% to 95% acetonitrile with 0.1% formic acid in water and 15 min at 95% acetonitrile with 0.1% formic acid in water at flow rates of 0.15 μl/min. Mass spectrometry was performed by Q Exactive plus mass spectrometer (Thermo) in a positive mode using repetitively full MS scan followed by collision induces dissociation (HCD) of the 10 most dominant ions selected from the first MS scan. The mass spectrometry data was analyzed using Proteome Discoverer 1.4 software with Sequest (Thermo) and Mascot (Matrix Science) algorithms against human uniprot database with mass tolerance of 10 ppm for the precursor masses and 0.05 amu for the fragment ions. Oxidation on Met were accepted as variable modifications and carbamidomethyl on Cys was accepted as static modifications. Minimal peptide length was set to six amino acids and a maximum of two mis cleavages was allowed. Peptide- and protein-level false discovery rates (FDRs) were filtered to 1% using the target-decoy strategy. Semi quantitation was done by calculating the peak area of each peptide based its extracted ion currents (XICs) and the area of the protein is the average of the three most intense peptides from each protein. To validate the interaction between endogenous NF1 and CAPN1, A375 and 74T melanoma cells were lysed and immunoprecipiated as described above with NF1 or control antibody normal rabbit immunoglobulin G (IgG). A375 stably overexpressing NF1 were immunoprecipiated with CAPN1 antibody (C0355, Sigma) or mouse IgG1 (5415, Cell Signaling) as a control. Samples were washed five times with the lysis buffer and separated by SDS-PAGE followed by Western blot analysis with anti-NF1 and anti-CAPN1 antibodies.

### *In vitro* cleavage assay

30 μg of protein lysate of A375, 74T cells and 293T stably overexpressing NF1 were incubated with purified CAPN1 (Sigma) at various concentrations supplemented with 10 mM CaCl_2_ and incubated at 30°C for 1 hour. Samples were then subjected to SDS-PAGE separation and western blotting with anti-NF1, anti-α-tubulin (05829, Merck) and anti-GAPDH (MAB374, Merck). The identification of NF1 degradation product was done on 293T stably over expressing NF1 with a Flag-tag. Cells were lysed as described above and 10 mg of the protein lysates were taken for immunoprecipitation with anti-Flag M2 affinity agarose beads (Sigma) overnight at 4°C. Then the beads were washed five times with lysis buffer. NF1 protein was eluted from the Flag beads by incubation with 150 ng/μl 3X Flag peptide (Sigma), followed by gentle agitation for 30 min at 4°C and then collection of the supernatant after short centrifugation. Purified NF1 protein after the elution was subjected to degradation with 0.1 U of purified CAPN1 followed by SDS-PAGE analysis with the following antibodies: polyclonal anti-NF1, monoclonal anti-Flag M2-Peroxidase (HRP) (A8592, Sigma), monoclonal anti-NF1 C’ terminus (SAB4200524, Sigma), monoclonal anti-NF1 N’ terminus (SAB4200499, Sigma) The suspected degradation product at the size of 40 kDa was cut from the gel after staining with Imperial™ protein stain (Thermo Scientific) and analyzed by mass spectrometry as described above.

### Calpain inhibitor treatment

Melanoma cells were seeded in 6-well plates at 1-2 × 10^5^ cells per well the day before the treatment and then treated with the indicated concentration of Calpain inhibitor I – ALLN (Sigma) at the designated time points. Cell lysates were generated by direct lysis into 2XSDS and then subsequently analyzed by protein blotting using the following antibodies: anti-P-AKT (S473) (9271, Cell Signaling), anti-P-AKT-(T308) (4056, Cell Signaling), anti-AKT (9272, Cell Signaling), anti-p44/42 MAPK (ERK1/2) (4695, Cell Signaling) and anti-ERK1/ERK2 (617400, Zymed).

### Ras activation assay

2X 15 cm plates of A375 and 74T melanoma cells were treated with 50 μM of Calpain inhibitor I for 6 hours or DMSO as control. Ras-GTP levels were detected using a Ras activation kit, following the manufacturer's instructions (Merck). RAS-GTP activation was quantified by using Image Lab software (Bio-Rad).

### Quantitative real-time PCR

Total RNA was extracted from A375 and 74T melanoma cells after treatment with Calpain inhibitor I in the indicated concentrations for 6 hours following the manufacturer's protocol for the RNeasy Mini Kit (74104, QIAGEN). Total RNA was eluted in 30 μl diethylpyrocarbonate (DEPC)-treated distilled H_2_O. A total of 500 ng of total RNA was used for single-strand complementary DNA (cDNA) synthesis using Iscript Reverse Transcription Supermix for RT-qPCR (1708841, Biorad). 2 μl of cDNA after 1:10 dilution were taken for the PCR reaction with either NF1 primers or GAPDH primers (Supplementary Table 2) mixed with 2×Fast SYBR Green PCR mix at a final volume of 10 μl in triplicate (4385612, Applied Biosystems). qRT-PCR analysis was done using the ABI Step One Plus Real-Time PCR system (with a standard program of stage 1: 95°C for 20 s; stage 2: 40 cycles of 95°C for 3 s and 60°C for 30 s). Results were analyzed using Microsoft Excel.

### Growth assays

A375 and 74T cells were seeded into 96-well plates at 200 cells per well either in 2.5% or 10% serum-containing medium respectively and incubated for 7-10 days. Samples were analyzed every 24-48 hours by lysing cells in 50 μl 0.2% SDS/well and incubating for 2 hours at 37°C prior to addition of 150 μl/well of SYBR Green I solution (1:750 SYBR Green I (Invitrogen-Molecular Probes) diluted in dH_2_0).

### siRNA depletion of endogenous CAPN1

Specific siRNA pool (ON-Targetplus) designed using siRNA design program for human *CAPN1* was purchased from Dharmacon (Thermo Fisher Scientific). A mixture of five siRNAs was used to transiently deplete *CAPN1* in A375 and 74T melanoma cells lines. Using DharmaFECT transfection reagent #1 (specific for siRNA), melanoma cells were transfected with 100 nM siRNA On-target pool in the presence of OptiMEM-I medium. Cells were incubated for 72 hours post-transfection before checking the protein expression levels of CAPN1, NF1 and the MAPK/PI3K downstream signaling pathways by western blot.

### Lentiviral shRNA

Constructs for stable depletion of CAPN1 or NF1 were obtained from Open Biosystems. Lentiviral stocks were prepared as previously described [57]. A375 and 74T melanoma cell lines were infected with lentivirus encoding shRNA for each condition (vector and two independent shRNAs specific to human *CAPN1* or *NF1*). Selection and growth were done as previously described. The shRNA constructs used in this study were shCAPN1-1 (TRCN0000003559), shCAPN1-2 (TRCN0000003560), shNF1-4 (TRCN0000039714) and shNF1-7 (TRCN0000039717). Stably infected pooled clones were tested for knockdown efficiency by western blot and by proliferation assay as described above.

### EC_50_ determinations and proliferation assays

Melanoma cell lines were tested by seeding 96-well plates at 3,000 cells per well. The next day, Calpain inhibitor I was added at concentrations from 1.25 μM to 160 μM in three replicates, with DMSO as a negative control. After 72 hours, cell proliferation was assessed using the CellTiter-Glo Luminescent Cell Viability Assay (Promega). EC_50_ values were determined using GraphPad Prism. The effect of Calpain inhibition combined by MEK inhibition on cell proliferation was tested by adding increasing concentrations of MEK inhibitor Trametinib (GSK1120210) (SelleckChem) from 1 pg to 10 μM and a constant concentration of Calpain inhibitor I (6 μM for A375, 76T, 108T) and 4 μM for 74T. Cells were evaluated for viability after 72 hours as described above. Statistical analysis was performed using Microsoft Excel to generate *P* values to determine significance (Student *t* test).

### Mutual exclusivity and survival analyses

Mutual exclusivity was calculated using WEXT [35]. For NF1, we considered under expression (<1/3-quantile across melanoma samples) or missense mutation as an alteration, while for CAPN1, we considered over expression (>2/3-quantile across melanoma samples) or missense mutation as an alteration.

For the survival analysis we performed a Kaplan Meier analysis and Cox regression analysis. We used a log rank test, where the effect size was quantified by the difference in the area under the curve (ΔAUC). To control for potential confounders, we used the following stratified Cox proportional hazard model to check this association, while controlling for the effect of respective genes, cancer types, sex, age, race, genomic instability, and tumor stage:

h_g_(t, patient) ~ h_0g_(t) exp(β_1_ I(*A*, *B*) + β_2_*age* + β_3_*GII*),

where g is an indicator variable over all possible combinations of patients’ stratifications based on cancer-type, race, sex, and tumor stage. h_g_ is the hazard function (defined as the risk of death of patients per unit time), and h_0g_(t) is the baseline-hazard function at time t of the g^th^ stratification. The model contains three covariates: (i) I(*A*, *B*): indicator variable denoting whether the specified alterations in both genes NF1 and CAPN1 simultaneously occurred in the given sample, (ii) age: age of the patient, and (iii) genomic instability: genomic instability of the tumor [58]. The βs s are the regression coefficient parameters of the covariates, which quantify the effect of covariates on the survival. All covariates are quantile-normalized to N(0,1). The βs are determined by standard likelihood maximization of the model.

## SUPPLEMENTARY MATERIALS FIGURES AND TABLES





## Abbreviations

NF1: Neurofibromin 1
CAPN1: Calpain1
