# Correction: CAPN1 is a novel binding partner and regulator of the tumor suppressor NF1 in melanoma

**DOI:** 10.18632/oncotarget.26685

**Published:** 2019-02-12

**Authors:** Michal Alon, Rand Arafeh, Joo Sang Lee, Sanna Madan, Shelly Kalaora, Adi Nagler, Tereza Abgarian, Polina Greenberg, Eytan Ruppin, Yardena Samuels

**Affiliations:** ^1^ Molecular Cell Biology Department, Weizmann Institute of Science, Rehovot, Israel; ^2^ Center for Bioinformatics and Computational Biology, The University of Maryland, College Park, Maryland, USA; ^3^ Cancer Data Science Lab, National Cancer Institute, National Institute of Health, Bethesda, Maryland, USA

**This article has been corrected:** The correct Acknowledgments information is given below:

**ACKNOWLEDGMENTS**

The authors thank Tamar Ziv and Arie Admon (The Smoler Proteomics Center, Technion, Israel) for the proteomic analysis. The authors also thank Antonella Di Pizio and Masha Y. Niv (The Hebrew University of Jerusalem, Israel) for performing the structural analysis on predicted NF1 cleavage sites. The authors also thank The De Botton Protein Profiling institute of the Nancy and Stephen Grand Israel National Center for Personalized Medicine, Weizmann Institute of Science for identifying the proteolytic product of NF1. The work was supported by Minerva Foundation Grant.

Original article: Oncotarget. 2018; 9:31264-31277. https://doi.org/10.18632/oncotarget.25805

