# Supplementary material for: CAPN1 is a novel binding partner and regulator of the tumor suppressor NF1 in melanoma
Source: Oncotarget. 2018 Jul 27;9(58):31264–77. doi: 10.18632/oncotarget.25805 (PMC6101293; doi:10.18632/oncotarget.25805)
Supplement: Supplementary file 1 [file oncotarget-09-31264-s001.pdf]

CAPN1 is a novel binding partner and regulator of the tumor suppressor NF1 in melanoma

SUPPLEMENTARY MATERIALS

```
21359 NF1 HUMAN Neurofibromin OS=Homo sapiens
 MAAHRPVEWV QAVVSRFDEQ LPIKTGQQNT HTKVSTEHNK ECLINISKYK FSLVISGLTT
 ILKNVNNMRI FGEAAEKNLY LSQLIILDTL EKCLAGQPKD TMRLDETMLV KQLLPEICHF
 LHTCREGNQH AAELRNSASG VLFSLSCNNF NAVFSRISTR LQELTVCSED NVDVHDIELL
 QYINVDCAKL KRLLKETAFK FKALKKVAQL AVINSLEKAF WNWVENYPDE FTKLYQIPQT
 DMAECAEKLF DLVDGFAEST KRKAAVWPLQ IILLILCPEI IQDISKDVVD ENNMNKKLFL
 DSLRKALAGH GGSRQLTESA AIACVKLCKA STYINWEDNS VIFLLVQSMV VDLKNLLFNP
 SKPFSRGSQP ADVDLMIDCL VSCFRISPHN NQHFKICLAQ NSPSTFHYVL VNSLHRIITN
 SALDWWPKID AVYCHSVELR NMFGETLHKA VQGCGAHPAI RMAPSLTFKE KVTSLKFKEK
 PTDLETRSYK YLLLSMVKLI HADPKLLLCN PRKQGPETQG STAELITGLV QLVPQSHMPE
 IAQEAMEALL VLHQLDSIDL WNPDAPVETF WEISSQMLFY ICKKLTSHQM LSSTEILKWL
 REILICRNKF LLKNKQADRS SCHFLLFYGV GCDIPSSGNT SQMSMDHEEL LRTPGASLRK
 GKGNSSMDSA AGCSGTPPIC RQAQTKLEVA LYMFLWNPDT EAVLVAMSCF RHLCEEADIR
 CGVDEVSVHN LLPNYNTFME FASVSNMMST GRAALQKRVM ALLRRIEHPT AGNTEAWEDT
 HAKWEQATKL ILNYPKAKME DGQAAESLHK TIVKRRMSHV SGGGSIDLSD TDSLQEWINM
 TGFLCALGGV CLQQRSNSGL ATYSPPMGPV SERKGSMISV MSSEGNADTP VSKFMDRLLS
 LMVCNHEKVG LQIRTNVKDL VGLELSPALY PMLFNKLKNT ISKFFDSQGQ VLLTDTNTQF
 VEQTIAIMKN LLDNHTEGSS EHLGQASIET MMLNLVRYVR VLGNMVHAIQ IKTKLCQLVE
 VMMARRDDLS FCQEMKFRNK MVEYLTDWVM GTSNQAADDD VKCLTRDLDQ ASMEAVVSLL
 AGLPLQPEEG DGVELMEAKS QLFLKYFTLF MNLLNDCSEV EDESAQTGGR KRGMSRRLAS
 LRHCTVLAMS NLLNANVDSG LMHSIGLGYH KDLQTRATFM EVLTKILQQG TEFDTLAETV
 LADRFERLVE LVTMMGDQGE LPIAMALANV VPCSQWDELA RVLVTLFDSR HLLYQLLWNM
 FSKEVELADS MQTLFRGNSL ASKIMTFCFK VYGATYLQKL LDPLLRIVIT SSDWQHVSFE
 VDPTRLEPSE SLEENQRNLL QMTEKFFHAI ISSSSEFPPQ LRSVCHCLYQ ATCHSLLNKA
 TVKEKKENKK SVVSQRFPQN SIGAVGSAMF LRFINPAIVS PYEAGILDKK PPPRIERGLK
 LMSKILQSIA NHVLFTKEEH MRPFNDFVKS NFDAARRFFL DIASDCPTSD AVNHSLSFIS
 DGNVLALHRL LWNNQEKIGQ YLSSNRDHKA VGRRPFDKMA TLLAYLGPPE HKPVADTHWS
 SLNLTSSKFE EFMTRHQVHE KEEFKALKTL SIFYQAGTSK AGNPIFYYVA RRFKTGQING
 DLLIYHVLLT LKPYYAKPYE IVVDLTHTGP SNRFKTDFLS KWFVVFPGFA YDNVSAVYIY
 NCNSWVREYT KYHERLLTGL KGSKRLVFID CPGKLAEHIE HEQQKLPAAT LALEEDLKVF
 HNALKLAHKD TKVSIKVGST AVQVTSAERT KVLGQSVFLN DIYYASEIEE ICLVDENQFT
 LTIANQGTPL TFMHQECEAI VQSIIHIRTR WELSQPDSIP QHTKIRPKDV PGTLLNIALL
 NLGSSDPSLR SAAYNLLCAL TCTFNLKIEG QLLETSGLCI PANNTLFIVS ISKTLAANEP
 HLTLEFLEEC ISGFSKSSIE LKHLCLEYMT PWLSNLVRFC KHNDDAKRQR VTAILDKLIT
 MTINEKQMYP SIQAKIWGSL GQITDLLDVV LDSFIKTSAT GGLGSIKAEV MADTAVALAS
 GNVKLVSSKV IGRMCKIIDK TCLSPTPTLE QHLMWDDIAI LARYMLMLSF NNSLDVAAHL
 PYLFHVVTFL VATGPLSLRA STHGLVINII HSLCTCSQLH FSEETKQVLR LSLTEFSLPK
 FYLLFGISKV KSAAVIAFRS SYRDRSFSPG SYERETFALT SLETVTEALL EIMEACMRDI
 PTCKWLDQWT ELAQRFAFQY NPSLQPRALV VFGCISKRVS HGQIKQIIRI LSKALESCLK
 GPDTYNSQVL IEATVIALTK LQPLLNKDSP LHKALFWVAV AVLQLDEVNL YSAGTALLEQ
 NLHTLDSLRI FNDKSPEEVF MAIRNPLEWH CKQMDHFVGL NFNSNFNFAL VGHLLKGYRH
 PSPAIVARTV RILHTLLTLV NKHRNCDKFE VNTQSVAYLA ALLTVSEEVR SRCSLKHRKS
 LLLTDISMEN VPMDTYPIHH GDPSYRTLKE TQPWSSPKGS EGYLAATYPT VGQTSPRARK
 SMSLDMGQPS QANTKKLLGT RKSFDHLISD TKAPKRQEME SGITTPPKMR RVAETDYEME
 TQRISSSQQH PHLRKVSVSE SNVLLDEEVL TDPKIQALLL TVLATLVKYT TDEFDQRILY
 EYLAEASVVF PKVFPVVHNL LDSKINTLLS LCQDPNLLNP IHGIVQSVVY HEESPPQYQT
 SYLQSFGFNG LWRFAGPFSK QTQIPDYAEL IVKFLDALID TYLPGIDEET SEESLLTPTS
 PYPPALQSQL SITANLNLSN SMTSLATSQH SPGIDKENVE LSPTTGHCNS GRTRHGSASQ
 VQKQRSAGSF KRNSIKKIV
```

Supplementary Figure 1: Identification of CAPN1 cleavage site in NF1. The amino acid sequence of NF1 protein is presented. Green colored amino acid residues represent the peptides that were identified by mass spectrometry analysis. Red lines indicate predicted cleavage sites determined by software package of GPS-CCD (Calpain Cleavage Detector) of the proteolytic fragment.

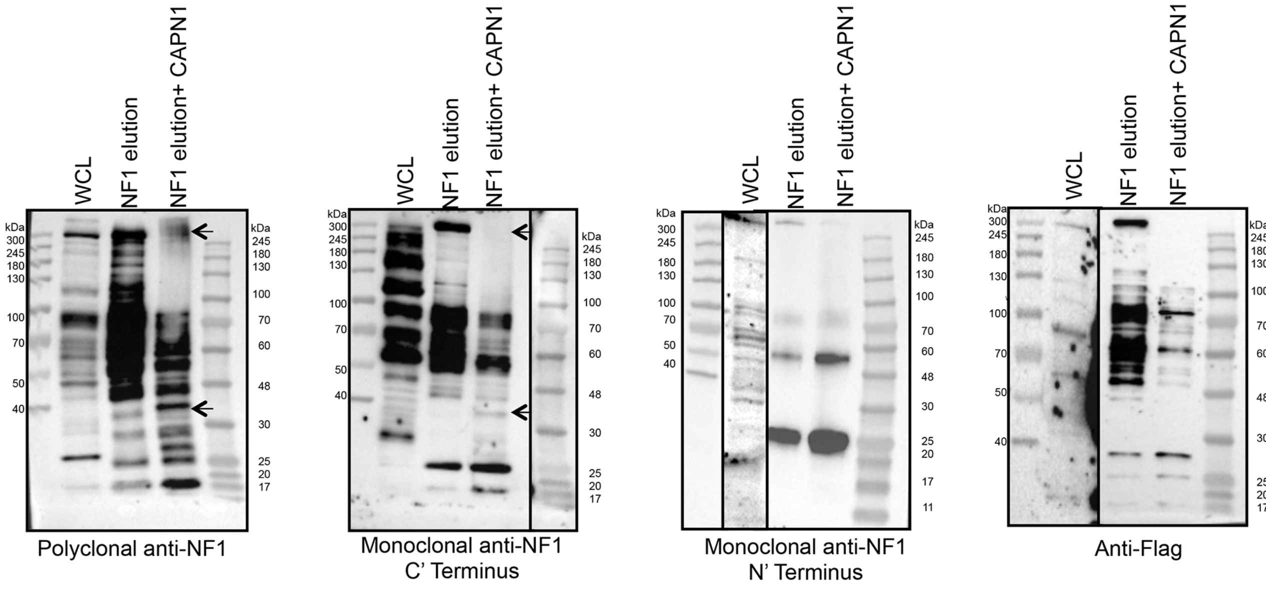

Supplementary Figure 2: Identification of NF1 degradation product by CAPN1 *in vitro*. NF1 was purified from 293T cells stably over expressing NF1 by Flag immunoprecipitation and elution by 3XFlag peptide. The purified NF1 protein was then subjected to CAPN1 degradation. The lysates were resolved by SDS-PAGE and blotted with the following antibodies: polyclonal anti-NF1, monoclonal anti C' terminus of NF1, monoclonal anti N' terminus of NF1 and anti Flag. The molecular weights are given in kilodaltons on both sides of each blot. The arrows indicate the detection of full length NF1 and of an approximately 40 kDa proteolytic fragment of NF1. The proteolytic product is only detected by the polyclonal anti-NF1 antibody and by the monoclonal anti-C' terminus of NF1.

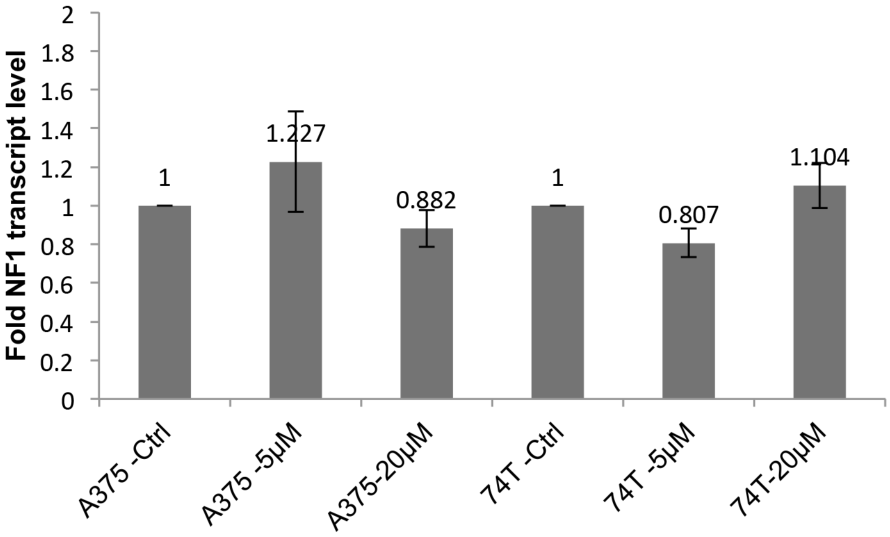

Supplementary Figure 3: CAPN1 inhibition does not alter NF1 transcript levels. qRT-PCR analysis of A375 and 74T cell lines after treatment with Calpain inhibitor I for 6 hours with the indicated concentrations or with DMSO as control. Error bars, s.e. n=2.

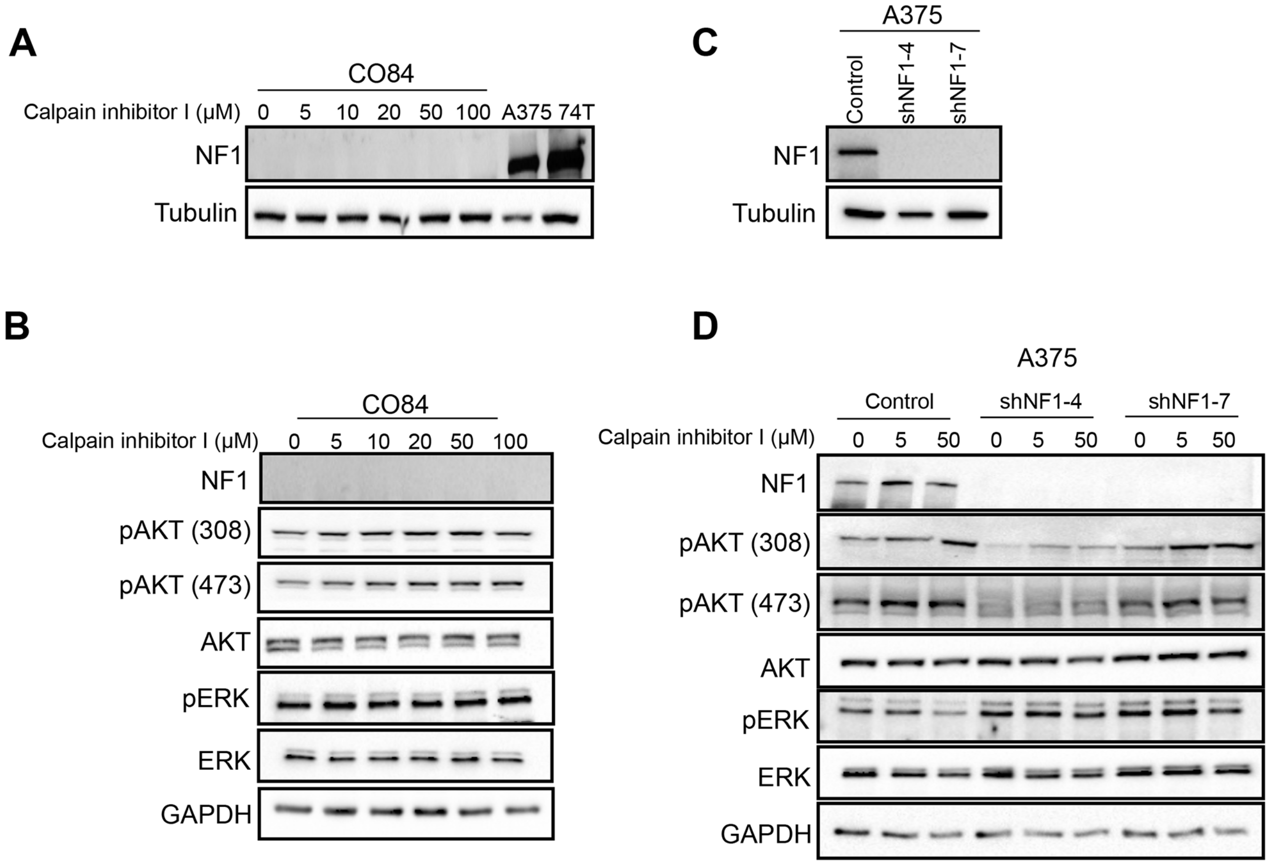

Supplementary Figure 4: CAPN1 inhibition does not stabilize NF1 levels and has no effect on RAS signaling in *NF1*-null cells. **(A)** The mutant *NF1* cell line CO84 was treated with increasing concentrations of Calpain inhibitor I (μM) for 6 hours or with DMSO as control, and NF1 levels were tested by immunoblot. **(B)** Cell lysates were analyzed by western blot with the indicated antibodies after treatment with increasing concentrations of Calpain inhibitor I. **(C)** Immunoblots of lysates generated from two shRNA mediated *NF1* knockdown (shNF1-4 and shNF1-7) compared to the control vector in A375 cells. **(D)** Immunoblot of lysates generated from shRNA mediated *NF1* knockdown and control in A375 after treatment with 5 and 50 μM of Calpain inhibitor I for 6 hours or with DMSO as control.

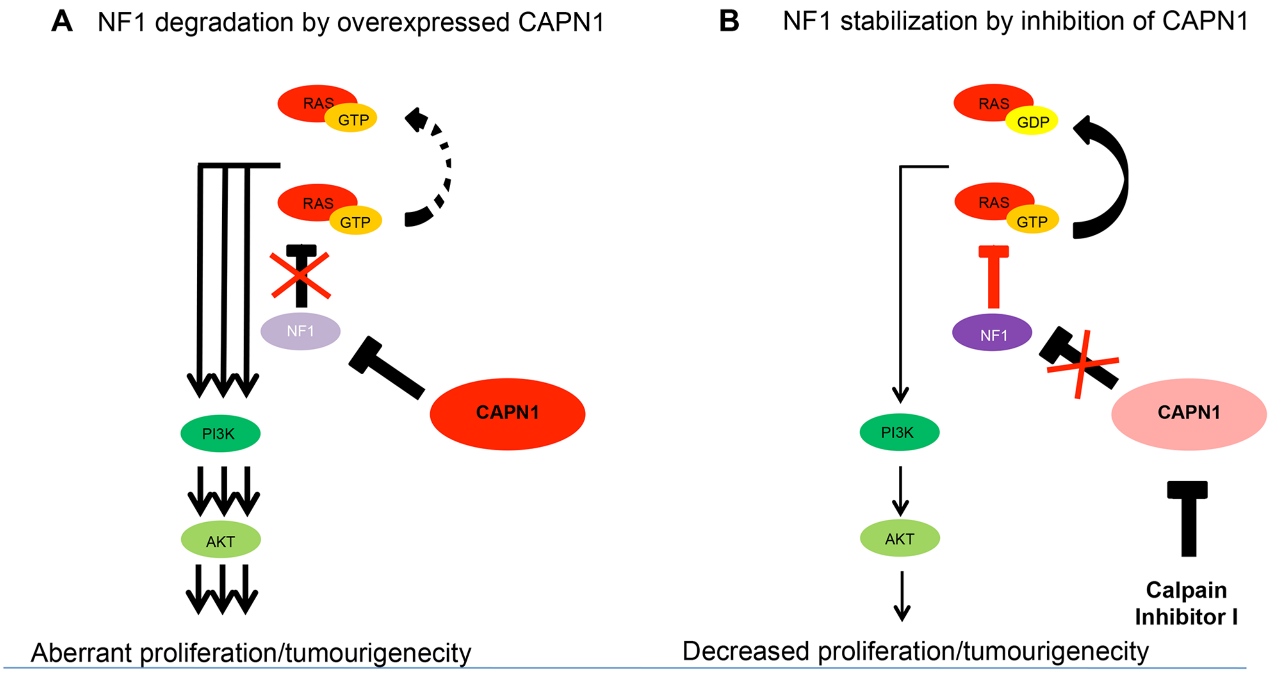

Supplementary Figure 5: The protease CAPN1 regulates the stabilization of NF1 in melanoma cells. (A) Excessive CAPN1 activity by overexpression or amplification in melanoma cells triggers the degradation of NF1. Since NF1 is a RasGAP, its degradation leads to RAS/AKT pathway activation, leading to aberrant proliferation and tumorigenecity. However, suppressing CAPN1 using a Calpain inhibitor I halts NF1 degradation and therefore NF1 is able to suppress RAS/AKT signaling, leading to decreased proliferation and tumorigenecity as seen in **(B)**.

Supplementary Table 1: Protein list identified by mass spectrometry of endogenous NF1 binding partners.

See Supplementary File 1

Supplementary Table 2: Sequences of qRT-PCR primers for NF1 and GAPDH amplification

Gene	Forward	Reverse
**NF1**	acgagtgtctcatgggcagat	actgttgtaagtgtcaggtccttttaag
**GAPDH**	ggagcgagatccctccaaaat	ggctgttgtcatacttctcatgg
